# Endoscopic management of acute cholecystitis in high‐risk surgical patients: A comprehensive review article

**DOI:** 10.1002/deo2.70133

**Published:** 2025-05-06

**Authors:** Akinori Maruta, Takuji Iwashita, Kensaku Yoshida, Shogo Shimizu, Masahito Shimizu

**Affiliations:** ^1^ Department of Gastroenterology Gifu Prefectural General Medical Center Gifu Japan; ^2^ First Department of Internal Medicine Gifu University Hospital Gifu Japan

**Keywords:** acute cholecystitis, endoscopic transpapillary gallbladder drainage, endoscopic ultrasound gallbladder drainage, high‐risk surgical patients, percutaneous transhepatic gallbladder drainage

## Abstract

Acute cholecystitis is frequently encountered in daily clinical practice, and early cholecystectomy is the standard therapy. In high‐risk surgical patients, such as those with advanced age, deteriorated performance status, or underlying diseases, conservative treatment is typically preferred to manage acute cholecystitis. However, in patients with a disease that is refractory to conservative treatment, drainage procedures are necessary to control the infection. At present, there are three basic approaches for gallbladder drainage: percutaneous transhepatic gallbladder drainage, endoscopic transpapillary gallbladder drainage, and endoscopic ultrasound gallbladder drainage. Each of these methods has advantages and disadvantages. Therefore, the appropriate treatment method is determined on a case‐by‐case basis, and no consistent strategy for gallbladder drainage has been established. This review aimed to summarize the characteristics of each drainage method and compare the clinical outcomes of the three procedures for acute cholecystitis in high‐risk surgical patients.

## INTRODUCTION

In the management of acute cholecystitis, early cholecystectomy is commonly performed. However, if the patient cannot withstand surgery, conservative treatment and biliary drainage should be considered.^1^ Currently, there are three basic approaches for gallbladder drainage: percutaneous transhepatic gallbladder drainage (PTGBD), endoscopic transpapillary GBD (ETGBD), and endoscopic ultrasound‐guided GBD (EUS‐GBD). PTGBD is a percutaneous external fistula management, whereas ETGBD and EUS‐GBD are endoscopic internal fistula management. Each procedure has advantages and disadvantages, and a drainage strategy for high‐risk surgical patients has not yet been established. This review summarizes the characteristics of each drainage method and compares the clinical outcomes of the three procedures for acute cholecystitis in high‐risk surgical patients.

### Percutaneous transhepatic GBD

**FIGURE 1:**
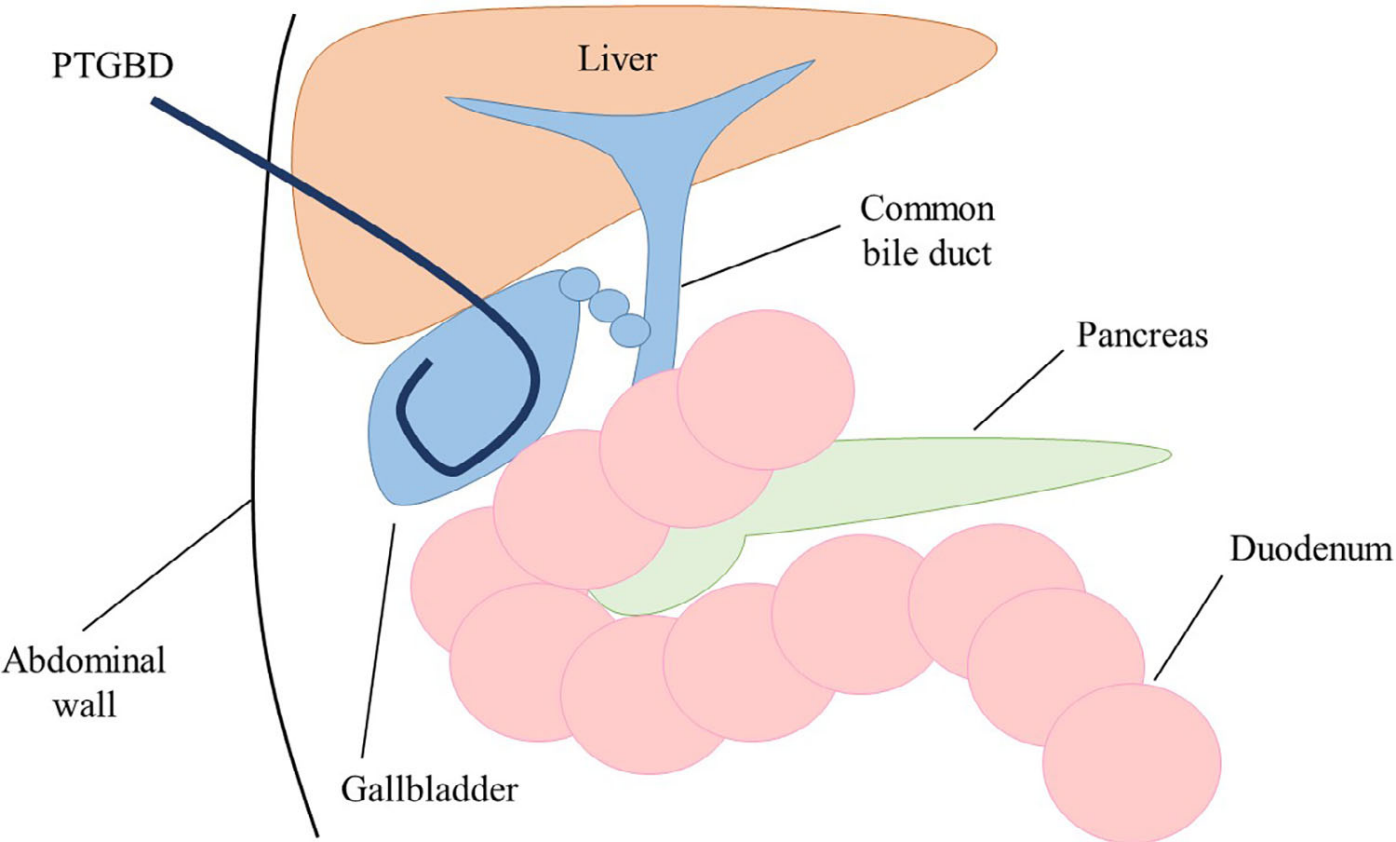
The schema of PTGBD. PTGBD, percutaneous transhepatic gallbladder drainage.

PTGBD is the traditional first‐line approach for infection control in patients with acute cholecystitis.^2^, ^3^ PTGBD was performed using the Seldinger technique. After an ultrasound‐guided transhepatic gallbladder puncture of the right intercostal space, an 8–10 Fr pigtail tube is placed over a guidewire under fluoroscopic guidance (Figure 1). PTGBD is a minimally invasive procedure that can be performed in a short time.^4^ It is associated with a high technical success rate (98%–99%) and clinical success rate (86%–97%) for the temporary decompression of the gallbladder.^5^, ^6^ PTGBD is recommended for the treatment of moderate to severe cholecystitis as it provides reliable drainage. The absence of a requirement for endoscope insertion or a surgical procedure minimizes the impact on cardiorespiratory status, which is beneficial for patients with unstable cardiorespiratory conditions, such as those in shock. Furthermore, external fistula management allows drainage monitoring and bile culture testing can be performed as necessary. Although PTGBD is an effective treatment for cholecystitis, some patients have contraindications for PTGBD as this procedure is associated with a bleeding tendency in patients taking antithrombotic agents and in those with thrombocytopenia, massive ascites, or an anatomically inaccessible location, such as that in patients with Chilaiditi's syndrome.^7^ PTGBD can cause discomfort or pain due to the external drainage tube and can negatively affect the patient's quality of life (QOL) due to limitations in activities of daily living. Typically, the tube cannot be removed until the maturation of the fistula to reduce the risk of bile leakage, which may lead to extended hospital stays for some patients. In addition, recurrence of cholecystitis after PTGBD removal occurs in 22%–47% of patients.^8^, ^9^, ^10^ If patients in whom the tube cannot be removed, long‐term placement may be necessary, though this significantly reduces the patient's QOL.

### Endoscopic transpapillary GBD

**FIGURE 2:**
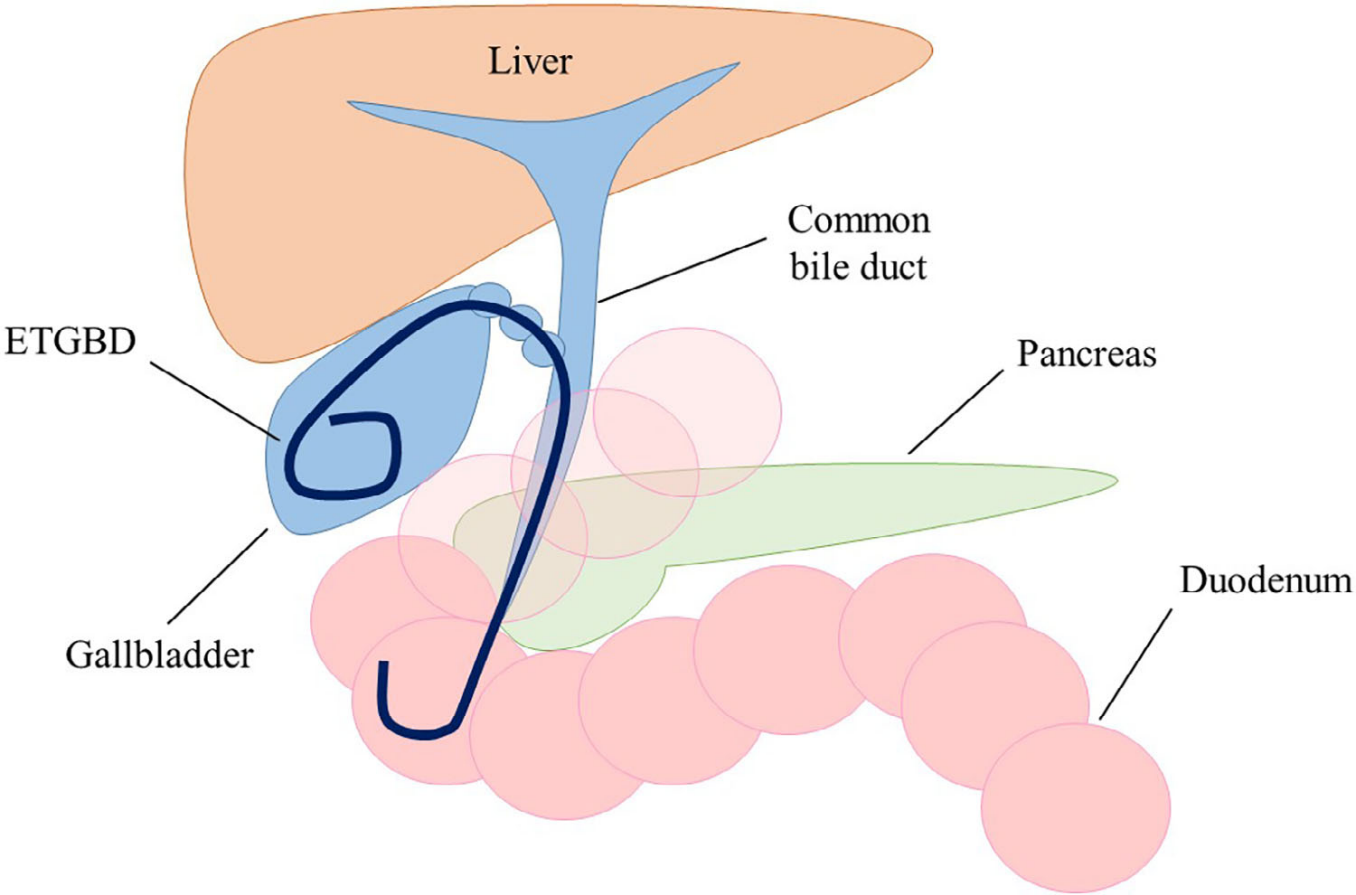
The schema of ETGBD. ETGBD, endoscopic transpapillary gallbladder drainage.

ETGBD has emerged as an alternative to PTGBD. It has been reported to be an effective and safe management method for acute cholecystitis in high‐risk surgical patients. Cholangiography is performed to evaluate the bile duct shape and bifurcation variation via endoscopic retrograde cholangiopancreatography (ERCP). After the successful advancement of the guidewire into the gallbladder, a 5–7 Fr drainage tube or plastic stent with a pigtail shape is inserted into the gallbladder (Figure 2). ETGBD does not require invasive procedures and can be performed safely, even in patients taking antithrombotic drugs. A retrospective multicenter study by Sagami et al. evaluated 130 patients who underwent ETGBD for acute cholecystitis.^11^ The patients were divided into an antithrombotic therapy (ATT) group (continuation of ATT on the day of the procedure and/or heparin substitution; *n* = 83) and a non‐ATT group (discontinuation or no use of ATT; *n* = 47). No bleeding adverse events (AEs) occurred in either group and the overall early AE rate was 3.1% (4/130; mild pancreatitis, *n* = 3 and cholangitis, *n* = 1). Therefore, ETGBD may be an ideal drainage method for patients with acute cholecystitis who take ATT.

### Endoscopic nasogallbladder drainage and endoscopic gallbladder stenting

ETGBD involves endoscopic nasogallbladder drainage (ENGBD) and endoscopic gallbladder stenting (EGBS). ENGBD is an external fistula management technique; therefore, it is possible to monitor the drainage and the risk of tube occlusion is low. In contrast, EGBS is an internal fistula management technique, and the burden on patients is low. ENGBD and EGBS are associated with shorter hospital stays for high‐risk surgical patients as they do not require fistula maturation. Itoi et al. conducted a prospective randomized controlled trial to compare the clinical outcomes of ENGBD (*n* = 37) and EGBS (*n* = 36) in patients with acute cholecystitis and observed no significant difference between the technical success rate (91.9% vs. 86.1%), mean procedure times (20.3 ± 12.1 vs. 22.2 ± 14.5 min), clinical success rate (94.1% vs. 90.3%), or AE (5.4% vs. 2.7%).^12^ However, the mean visual analog score of post‐procedure pain in the ENGBD group was significantly higher than that in the EGBS group (1.3 ± 1.1 vs. 0.4 ± 0.8, respectively; *p* < 0.001). These results suggest that ENGBD and EGBS have equivalent clinical outcomes for acute cholecystitis and that EGBS may reduce the burden on patients.

### Factors associated with technical failure in ETGBD and their improvement measures

The greatest challenge of ETGBD is its technical success rate. The pooled technical success rate of ETGBD is 80.9% (95% confidence interval, 74.7%–86.2%), which is lower than that reported for PTGBD (nearly 100%).^13^ Several hurdles need to be overcome to accomplish ETGBD, including cystic duct cannulation, cystic duct guidewire insertion into the gallbladder, and drainage tube or stent insertion into the gallbladder. Several previous studies have investigated the factors related to technical failure in ETGBD (Table 1).^14–18^ The factors predicting technical success based on patient characteristics and imaging findings before treatment have been studied.^16^ A total of 323 patients underwent ETGBD for the management of acute cholecystitis, and a technical success rate of 72.8% was achieved. The technical success rates in each region of the cystic duct were as follows: proximal/distal, 65.9%/93.6%; right/left:74.0%/65.2%; and cranial/caudal, 83.5%/20.0%. In both univariate and multivariate analysis, the presence of cystic duct stone, dilation of the common bile duct (CBD), and cystic duct direction (proximal and caudal branches) were identified as significant factors affecting the technical failure of ETGBD. Hirakawa et al. conducted a retrospective study to identify the factors associated with the technical failure of ETGBD by focusing on clinical characteristics, anatomical features, and procedural factors.^18^ In 182 patients who underwent ETGBD, the technical success rate was 84.6%. Univariate and multivariate analyses identified the right cranial direction and spiral‐type course of the cystic duct as significant anatomical features and cystic duct injury as a significant procedural feature contributing to the technical failure of ETGBD. These results suggest that patient factors also have a strong influence on the technical failure of ETGBD. Therefore, in patients with characteristics that have been identified as risk factors for technical failure, it may be better to consider other drainage methods to prevent ERCP‐related AEs.

**TABLE 1:** Factors associated with technical failure of endoscopic transpapillary gallbladder drainage.

Authors	Study design	No. of patients	Technical success	Patient factors	Procedural factors
Ogawa et al., 2008^13^	Retrospective	11	63.6%	•Minor‐axis length of gallbladder •Wall thickness of the gallbladder	
Yane et al., 2015^14^	Prospective	27	77.7%	•Older age •Wall thickness of the gallbladder	
Maruta et al., 2020^15^	Retrospective	323	72.8%	•Proximal branch of the cystic duct •Caudal direction of the cystic duct •Cystic duct stones •Dilation of CBD	
Sato et al., 2023^16^	Retrospective	242	87%		•Cystic duct injury
Hirakawa et al., 2024^17^	Retrospective	182	84.6%	•Right caudal direction of the cystic duct •Spiral‐type course of the cystic duct	•Cystic duct injury

Abbreviations: CBD, common bile duct.

### Methods to improve the technical success rate of ETGBD

Various approaches have been proposed to improve the technical success rate of ETGBD.^19^, ^20^, ^21^, ^22^, ^23^ Sagami et al. compared the efficacy of ETGBD using intraductal ultrasonography (IDUS) with that of using ETGBD alone.^19^ A total of 100 consecutive patients with acute cholecystitis who required ETGBD were retrospectively recruited. The first 50 consecutive patients were treated with ETGBD without IDUS, and the next 50 patients were treated with ETGBD with IDUS. The technical success rate of ETGBD with IDUS was significantly higher than that of ETGBD without IDUS (92.0% vs. 76.0%, *p *= 0.044). No significant difference in procedure length (74.0 min vs. 66.7 min, *p *= 0.310) was observed between the two groups. ETGBD combined with IDUS is expected to make it easier to identify the cystic duct during cannulation, without requiring significant additional time. Another retrospective study by Yoshida et al. investigated 101 patients who underwent ETGBD.^20^ The technical success rate of conventional ETGBD (C‐ETGBD) and cholangioscopic‐assisted (SpyGlass DS) ETGBD (SG‐ETGBD) were evaluated. C‐ETGBD was successful in 73 patients (72.3%). SG‐ETGBD was successful in 11 of 13 patients (84.6%) who had C‐ETGBD failure. Optional SG‐ETGBD significantly increased the final success rate (94.1%) compared with C‐ETGBD alone (*p *= 0.003). SG‐ETGBD worked as an excellent troubleshooter when the cystic duct orifice could not be identified or the guidewire could not be advanced across the downturned angle of the cystic duct. These results indicate that the combined use of IDUS or cholangioscopy can assist in identifying the cystic duct branch and advancing a guidewire into the cystic duct, potentially improving the technical success rate of ETGBD.

### Endoscopic ultrasound GBD

**FIGURE 3:**
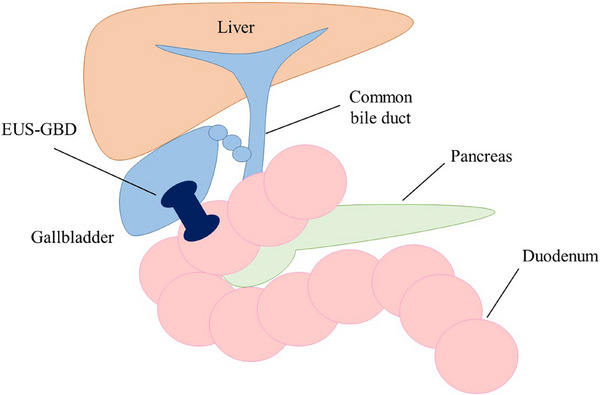
The schema of EUS‐GBD. EUS‐GBD, endoscopic ultrasound‐guided gallbladder drainage.

EUS‐GBD is a novel procedure for the treatment of acute cholecystitis (Figure 3). The gallbladder is visualized by a linear echoendoscope and a suitable puncture site from the stomach or the duodenum without intervening blood vessels is located. In the conventional method, the gallbladder is punctured with a 19‐gauge needle, and a guidewire is passed through the needle and looped in the gallbladder. After the fistula dilation with a bougie dilator or balloon catheter, a double‐pigtail plastic stent or covered self‐expandable metallic stent is placed across the fistula. In the direct method, the delivery system of the cautery‐enhanced lumen apposing metal stent (LAMS) is directly inserted into the gallbladder without prior needle insertion. The distal flange of the stent is deployed under EUS guidance, followed by the deployment of the proximal flange under endoscope guidance. Recently, even in high‐risk surgical patients, surgical indications after improvement of cholecystitis by gallbladder drainage have been proposed. The presence of a LAMS precluded a minimally invasive surgical approach and necessitated conversion to open or subtotal cholecystectomy. Therefore, EUS‐GBD should be reserved for a selective cohort of never‐surgery patients and not for all patients broadly stratified as high‐risk surgical candidates.^24^ Although EUS‐GBD has high technical and clinical success rates, ensuring its safety remains a top priority. As the gallbladder and digestive tract are anatomically separate organs, there are concerns regarding procedure‐related AEs including bile leakage and stent migration.

### Verification of the efficacy and safety of EUS‐GBD

Several studies have aimed to verify and improve the safety of EUS‐GBD. Grover et al. conducted a comprehensive literature review to evaluate clinical outcomes of the transgastric (TG) and transenteric (transduodenal [TD]/transjejunal [TJ]) approaches in EUS‐GBD.^25^ In total, 15 studies reporting the TD/TJ approach and nine using the TG approach were included. There were no significant differences in the technical success (TG vs. TD/TJ: 91.3% vs. 95.3%, *p *= 0.22) or clinical success (83.3% vs. 91.7%, *p *= 0.16) rates. However, the rate of AEs was significantly higher in the TG group (27.5% vs. 15.2%; *p *= 0.07). The commonly noted AEs with EUS‐GBD include stent migration, stent occlusion, biliary peritonitis, pneumoperitoneum, and recurrence of cholecystitis due to food impaction. Compared to the stomach, the wall of duodenum/jejunum has less peristaltic activity which may decrease the risk of stent migration and stent occlusion due to tissue overgrowth. The potential for reflux of food contest into the gallbladder may be lesser with the TD approach resulting in reduced risk of stent occlusion or infection related to reflux.^26^, ^27^ Therefore, the TD/TJ approach may be associated with a more favorable AE profile with equal efficacy to the TG approach for EUS‐GBD. Another retrospective study by Anderloni et al. compared the clinical outcomes of EUS‐GBD according to stent type.^28^ Twenty‐one studies investigated a total of 166 patients who underwent EUS‐GBD for the management of acute cholecystitis using different types of stents. The pooled technical success rates for plastic stents, SEMS, and LAMS were 100%, 98.6%, and 91.5%, respectively, and the pooled clinical success rates were more than 90% for all types of stents (100%, 95.5%, and 90.1% for plastic stents, SEMS, and LAMS, respectively). The success rates were high and comparable among the different types of stents. However, the rate of AEs was 18.2% in the plastic stent group, 12.3% in the SEMS group, and 9.9% in the LAMS group. Therefore, the use of LAMS during EUS‐GBD has high potential in terms of efficacy and safety.

### Clinical outcomes of EUS‐GBD with LAMS

Several studies have reported the efficacy and safety of EUS‐GBD using LAMS in high‐risk surgical patients. Dollhopf et al. retrospectively examined 75 high‐risk surgical patients who underwent EUS‐GBD with LAMS for acute cholecystitis.^29^ The technical and clinical success rates were 98.7% and 95.9%, respectively. The rate of procedure‐related AEs was 2.6% (2/75): one patient experienced perforation requiring surgery and one patient experienced major bleeding that resolved conservatively. The total mean procedural time was 26 min (range, 8–60 min), whereas the mean stent deployment time was 4.5 min (range, 1–20 min). These results show that EUS‐GBD using LAMS has a very good safety profile with high technical and clinical success rates. A study by Martinez‐Moreno et al. investigated 50 high‐risk surgical patients who underwent EUS‐GBD for acute cholecystitis to analyze the 3‐year long‐term outcomes of EUS‐GBD with LAMS.^30^ AEs occurred in 18%, 20%, and 26% of patients in the first, second, and third years, respectively. Recurrence of cholecystitis recurred in two patients (4%). Seven stent migrations (14%) occurred, though all were asymptomatic. The symptomatic LAMS‐related AEs (37.5%) were related to a gastric location of the stent compared with a duodenal location (66.7% vs. 12.5%, *p *= 0.03). No stent‐related bleeding or mortality was observed. There is no established consensus regarding the strategy after LAMS placement in EUS‐GBD for acute cholecystitis in high‐risk surgical patients. In several studies, permanent LAMS placement was required due to patients' poor general condition,^29–32^ however, Teoh et al. have reported that a regular endoscope was inserted through the gallbladder stent into the gallbladder to check for the presence of gallstones, and if all gallstones were cleared, the LAMS was removed and replaced with a permanent 7 Fr double pigtail plastic catheter.^33^ Based on these results, EUS‐GBD using LAMS is effective and safe in both the short‐ and long‐terms for the management of acute cholecystitis in high‐risk surgical patients.

### Verification of a comparative study of three drainage procedures

Each gallbladder drainage procedure has advantages and disadvantages. The characteristics of each procedure are summarized in Table 2. Drainage strategies for acute cholecystitis in high‐risk surgical patients have not yet been established, although the results of various comparative studies have been reported.

**TABLE 2:** The characteristics of percutaneous transhepatic gallbladder drainage, endoscopic transpapillary gallbladder drainage, and endoscopic ultrasound‐guided gallbladder drainage.

	PTGBD	ETGBD	EUS‐GBD
Pros	•Standard treatment •Technical success •Short procedure time •Bile monitoring	•Internal fistula •Permanent placement •Simultaneous bile duct evaluation and treatment •No fistula maturation required	•Internal fistula •Technical success •Permanent placement •No fistula maturation required
Cons	•Needle puncture required •External fistula •Fistula maturation period required •Inapplicable case (ascites, bleeding tendency, anatomically)	•Endoscope insertion required •Technical success •Endoscopic related AEs	•Endoscope insertion required •Bile leak •Stent migration •Few dedicated devices •Inapplicable case (ascites, bleeding tendency, anatomically)

Abbreviations: ETGBD, endoscopic transpapillary gallbladder drainage; EUS‐GBD, endoscopic ultrasound‐guided gallbladder drainage; PTGBD, percutaneous transhepatic gallbladder drainage.

### Short‐term outcomes of PTGBD versus ETGBD

There are several reports comparing the clinical outcomes of PTGBD and ETGBD for the management of acute cholecystitis.^34^, ^35^ A retrospective study by Iino et al. compared the efficacy and safety of PTGBD and ETGBD in patients with acute cholecystitis.^34^ ETGBD and PTGBD were successfully performed in 33 patients (77%) and 42 patients (100%; *p *< 0.001), respectively, with no significant difference in the occurrence of AEs between the groups. After propensity score matching, no significant differences in improvement of white blood cell count or serum C‐reactive protein level were observed. However, the length of hospitalization was significantly shorter among patients who underwent ETGBD than among those treated with PTGBD. Multivariate logistic regression analysis revealed ETGBD (odds ratio, 7.07; 95% confidence interval, 2.22–22.46) as an independent factor associated with the hospitalization period. These results suggest that ETGBD was as useful as PTGBD and more effective than PTGBD in reducing the length of the hospital stay because ETGBD did not require a period of fistula maturation.

### Long‐term outcomes of PTGBD versus ETGBD

Permanent EGBS is a treatment option for high‐risk surgical patients and there have been several reports regarding its usefulness and safety.^36^, ^37^, ^38^, ^39^, ^40^, ^41^, ^42^ The results of a comparative study of the long‐term outcomes of permanent EGBS and the removal of gallbladder drainage devices in high‐risk surgical patients with acute cholecystitis are summarized in Table 3. A total of 180 high‐risk surgical patients with acute cholecystitis were retrospectively divided into two groups: the EGBS group (long‐term placement of EGBS) and the removal group (removal of the drainage tube after PTGBD or ENGBD). The cumulative late AE rates were 5.0% and 22.1% in the EGBS and removal group (*p *= 0.002), respectively, with a median follow‐up period of 375 and 307 days, respectively. The cumulative cholecystitis recurrence rates were 5.0% (2/40) in the EGBS group and 16% (21/131) in the removal group (*p *= 0.024). A multicenter comparative study by Inoue et al. evaluated 528 high‐risk surgical patients with acute cholecystitis to examine the long‐term effects of EGBS. The 1‐, 3‐, and 5‐year cumulative recurrence rates of cholecystitis were 3.8%, 7.2%, and 7.2%, respectively, in the EGBS group, and 11.7%, 17.6%, and 30.2%, respectively, in the PTGBD group (*p *= 0.001). In contrast, the rates of symptomatic late AEs (excluding recurrence of cholecystitis) were 8.2%, 22.7%, and 31.4%, respectively, in the EGBS group and 7.5%, 10.9%, and 13.1%, respectively, in the PTGBD group (*p *= 0.035). The 1‐, 3‐, and 5‐year cumulative incidence rates of overall late AE were 12.0%, 30.4%, and 40.4%, respectively, in the EGBS group and 19.2%, 28.3%, and 42.5%, respectively, in the PTGBD group (*p *= 0.649). Therefore, permanent EGBS in high‐risk surgical patients with acute cholecystitis is considered effective as it is associated with a decreased risk of late AEs, including the recurrence of cholecystitis, though the frequency of other late AEs increased as the indwelling period increased.

**TABLE 3:** Studies comparing long‐term outcomes of endoscopic gallbladder stenting and removal of drainage.

Author	Study design	Drainage	No. of patients	Drainage tube /stent	Follow‐up period (median/mean)		Recurrent cholecystitis		Late AE (including recurrent cholecystitis)	
Kedia et al., 2015^31^	Retrospective	EGBS	30	5 or 7 Fr Pigtail	8.9 M	*p =* 0.39	‐	‐	0%	*p* < 0.0001
		PTGBD	43	8 or 10 Fr	9.4 M		‐		27.9%	
Inoue et al., 2016^32^	Retrospective	EGBS	35	7 Fr Pigtail	473 D	*p =* 0.649	0%	*p =* 0.043	9.1%	*p =* 0.207
		PTGBD / PTGBA	29	7 or 8.5 Fr	485 D		17.2%		24.1%	
Maruta et al., 2021^33^	Retrospective	EGBS	40	5 or 6 Fr Pigtail	375 D	*p =* 0.577	5.0%	*p =* 0.024	5.0%	*p =* 0.002
		PTGBD / ENGBD	131	PTGBD: 8 or 8.5 Fr ENGBD: 5 or 6 Fr Pigtail	307 D		16.0%		22.1%	
Inoue et al., 2023^35^	Retrospective	EGBS	158	7 Fr Pigtail	1115.5 D	*p =* 0.678	6.3%	*p =* 0.001	25.9%	*p =* 0.500
		PTGBD	120	7 or 8.5 Fr	1136.2 D		19.2%		30.0%	

Abbreviations: AE, adverse event; EGBS, endoscopic gallbladder stenting; ENGBD, endoscopic nasogallbladder drainage; PTGBA, percutaneous transhepatic gallbladder aspiration; PTGBD, percutaneous transhepatic gallbladder drainage.

### Comparative studies of EUS‐GBD versus PTGBD and EUS‐GBD versus ETGBD

Several studies have compared the clinical outcomes of EUS‐GBD and PTGBD for acute cholecystitis in high‐risk surgical patients.^33^, ^43^, ^44^, ^45^, ^46^ Teoh et al. conducted an international randomized multicenter controlled trial to compare EUS‐GBD with LAMS (*n* = 39) with PTGBD (*n* = 40) as the definitive treatment for acute cholecystitis in high‐risk surgical patients.^33^ Technical success rates (97.4% vs. 100%, *p *= 0.494) and clinical success rates (92.3% vs. 92.5%, *p *= 1) were statistically similar, indicating high therapeutic efficacy. EUS‐GBD significantly reduced the rate of AEs at one year (25.6% vs. 77.5%, *p *< 0.001) and 30 days (12.8% vs. 47.5%, *p *= 0.010), the rate of re‐interventions after 30 days (2.6% vs. 30%, *p *= 0.001), the number of unplanned readmissions (15.4% vs. 50%, *p *= 0.002), and the rate of recurrent cholecystitis (2.6% vs. 20%, *p *= 0.029). The postprocedural pain scores and analgesic requirements were also lower (*p *= 0.034). A systematic review and meta‐analysis by Hayat et al. compared the safety of EUS‐GBD with LAMS (*n* = 298) and PTGBD (*n* = 412) for acute cholecystitis in high‐risk surgical patients as reported in six previous studies.^43^ Both EUS‐GBD and PTGBD had similar short‐term AEs; however, EUS‐GBD was associated with a lower rate of delayed AEs (odds ratio [OR], 0.21; 95% CI, 0.07–0.61; *p* ≤ 0.01) and overall AEs (OR, 0.43; 95% CI, 0.30–0.61; *p* ≤ 0.01). These results show that EUS‐GBD using LAMS for acute cholecystitis in high‐risk surgical patients is effective and appears to be safer than PTGBD in terms of long‐term outcomes.

Similarly, several studies compared EUS‐GBD with ETGBD for the treatment of acute cholecystitis in high‐risk surgical patients.^47^, ^48^, ^49^ Inoue et al. retrospectively examined 379 high‐risk surgical patients who underwent ETGBD or EUS‐GBD using SEMS or plastic stents for acute cholecystitis.^48^ After propensity score‐matching, the technical success rate of EUS‐GBD was significantly higher than that of ETGBD (96.7% vs. 78.9%, *p *< 0.001). The rate of symptomatic late AEs, in addition to cholecystitis, was significantly lower in the EUS‐GBD group than in the ETGBD group (1.3% vs. 13.4%; *p *= 0.006). Multivariate analysis revealed that EUS‐GBD was associated with a significantly longer time to late AE (hazard ratio [HR], 0.26; 95% CI, 0.10–0.67; *p *= 0.005). A systematic review and meta‐analysis by Krishnamoorthi et al. compared the effectiveness and safety of EUS‐GBD (*n* = 259) and ETGBD (*n* = 598) for acute cholecystitis in high‐risk surgical patients in five studies.^49^ EUS‐GBD was associated with higher technical [pooled OR 5.22 (95% CI 2.03–13.44; *p *= 0.0006] and clinical success [pooled OR 4.16 (95% CI 2.00–8.66; *p *= 0.0001)] rates than ETGBD. There was no statistically significant difference in the rate of overall AEs [pooled OR 1.30 (95% CI 0.77–2.22; *p *= 0.33)]. EUS‐GBD was associated with a lower rate of recurrent cholecystitis [pooled OR 0.33 (95% CI 0.14–0.79; *p *= 0.01)]. These studies indicate that EUS‐GBD resulted in a higher technical success rate and a lower late AE rate, including recurrent cholecystitis. Therefore, EUS‐GBD may be more suitable than ETGBD for the endoscopic treatment of acute cholecystitis in high‐risk surgical patients.

### Comparative study of PTGBD versus ETGBD versus EUS‐GBD

Several studies have compared the clinical outcomes of PTGBD, ETGBD, and EUS‐GBD for acute cholecystitis in high‐risk surgical patients.^5^, ^50^, ^51^, ^52^, ^53^ The results of each study are summarized in Table 4. Siddiqui et al. conducted an international, multicenter, retrospective study to evaluate the clinical outcomes of PTGBD, ETGBD, and EUS‐GBD for acute cholecystitis in high‐risk surgical patients.^5^ A total of 372 patients who underwent gallbladder drainage, including 146 who underwent PTGBD, 124 who underwent ETGBD, and 102 who underwent EUS‐GBD with LAMS). The technical (PTGBD: 98%, EUS‐GBD: 88%, ETGBD: 94%; *p *= 0.004) and clinical (PTGBD: 97%, EUS‐GBD: 80%, ETGBD: 90%; *p *< 0.001) success rates were significantly higher in the PTGBD and EUS‐GBD groups than those in the ETGBD group. The rates of early AEs were not significantly different between the groups (*p *= 0.07), though late AEs were less frequent in the EUS‐GBD group than in the ETGBD and the PTGBD groups [1.9% vs. 4.8% vs. 19.8%, respectively; *p *< 0.001). Patients in the EUS‐GBD and ETGBD groups required fewer unplanned hospital readmissions than those in the PTGBD group (4% vs. 3.2% vs. 19.8%, respectively; *p *< 0.001). The mean length of hospital stay in the EUS‐GBD group was significantly shorter than that in the ETGBD and PTGBD groups (16 vs. 18 vs. 19 days, respectively; *p *= 0.01), whereas the rate of additional surgical interventions was significantly higher in the PTGBD group compared to the EUS‐GBD and ETGBD groups (49% vs. 4% vs. 11%, respectively; *p *< 0.0001). A systematic review and network meta‐analysis by Podboy et al. compared three drainage methods for acute cholecystitis in high‐risk surgical patients.^53^ In ten studies including 1,267 patients (493 who underwent PTGBD, 302 who underwent ETGBD, and 472 who underwent EUS‐GBD) were investigated. PTGBD and EUS‐GBD had the highest likelihood of technical success (PTGBD vs. ETGBD vs. EUS‐GBD: 1.02 vs. 2.98 vs. 2.00) and clinical success (PTGBD vs. ETGBD vs. EUS‐GBD: 1.55 vs. 2.98 vs. 1.48). EUS‐GBD was associated with the lowest risk of recurrent cholecystitis (PTGBD vs. ETGBD vs. EUS‐GBD: 2.02 vs. 2.891 vs. 1.089). PTGBD was associated with the highest risk of reintervention (PTGBD vs. ETGBD vs. EUS‐GBD: 2.99 vs. 1.199 vs. 1.81) and unplanned readmission (PTGBD vs. ETGBD vs. EUS‐GBD: 2.944 vs. 1.474 vs. 1.582). These results suggest that PTGBD and EUS‐GBD for the management of acute cholecystitis in high‐risk surgical patients result in higher technical success rates than ETGBD with no significant differences in the early AE rate. Furthermore, long‐term outcomes such as late AEs, unplanned readmission, and reinterventions, were poorer with PTGBD.

**TABLE 4:** Comparative studies of gallbladder drainage in high‐risk surgical patients.

Author	Study design	No. of patients	Technical success	Clinical success	Early AEs	Late AEs
Siddiqui et al., 2019^4^	Retrospective	PTGBD: 146 ETGBD: 124 EUS‐GBD: 102	98% 88% 94%	97% 80% 90%	4.1% 7.2% 11.8%	19.8% 4.8% 1.9%
Rerknimitr et al., 2020^44^	Review	‐ ‐ ‐	98%–99% 50%–100% 91.5%–100%	86%–97% 76.3%–97% 90.1%–100%	20%–20% 78.7%–10% 9.9%–18.2%	‐ ‐ ‐
Mohan et al., 2020^45^	Systematic review and meta‐analysis	PTGBD: 13 351 ETGBD: 1223 EUS‐GBD: 557	98.7% 83% 95.3%	89.3% 88.1% 96%	15.1% 9.6% 12.4%	^※^10.8% ^※^4.6% ^※^4.2%
Podboy et al., 2021^47^	Systematic review and meta‐analysis	PTGBD: 493 ETGBD: 302 EUS‐GBD: 472	PTGBD versus EUS‐GBD: RR, 1.041 [95% CI, 1.005–1.09] ETGBD versus EUS‐GBD: RR, 0.8087 [95% CI, .4333–0.9933] ETGBD versus PTGBD: RR, 0.7775 [95% CI, .4164–0.9587]	PTGBD versus EUS‐GBD: RR, 0.9884 [95% CI, 0.8747–1.052] ETGBD versus EUS‐GBD: RR, 0.7508 [95% CI, 0.3806‐0.9671] ETGBD versus PTGBD: RR, 0.7600 [95% CI, 0.3962‐0.9844]	PTGBD versus EUS‐GBD: RR, 1.02 [95% CI, 0.424–1.911] ETGBD versus EUS‐GBD: RR, 1.16 [95% CI, 0.4222–2.383] ETGBD versus PTGBD: RR, 1.261 [95% CI, 0.4145–3.062]	^※^PTGBD versus EUS‐GBD: RR, 1.962 [95% CI, 0.750–4.09] ^※^ETGBD versus EUS‐GBD: RR, 3.72 [95% CI, 1.386–7.541] ^※^PTGBD versus ETGBD: RR, 2.201 [95% CI, 0.660–5.373]

Abbreviations: AEs, adverse events; ETGBD, Endoscopic transpapillary gallbladder drainage; EUS‐GBD, Endoscopic ultrasound gallbladder drainage; PTGBD, Percutaneous transhepatic gallbladder drainage; RR, risk ratio; 95% CI, 95% confidence interval.

^※^Not including other than recurrent cholecystitis.

## CONCLUSION

In conclusion, EUS‐GBD using LAMS may provide more favorable clinical outcomes than PTGBD or ETGBD in high‐risk surgical patients with acute cholecystitis. However, each drainage technique has advantages and disadvantages, and it is necessary to carefully consider the characteristics of each procedure and choose the most appropriate treatment strategy for patients on an individualized basis.

## CONFLICT OF INTEREST STATEMENT

None.

## References

[1] Okamoto K , Suzuki K , Takada T *et al*. Tokyo Guidelines 2018: Flowchart for the management of acute cholecystitis. J Hepatobiliary Pancreat Sci 2018; 25: 55–72. 29045062 10.1002/jhbp.516

[2] Itoi T , Tsuyuguchi T , Takada T *et al*. TG13 indications and techniques for biliary drainage in acute cholangitis (with videos). J Hepatobiliary Pancreat Sci 2013; 20: 71–80. 23307008 10.1007/s00534-012-0569-8

[3] Ito K , Fujita N , Noda Y *et al*. Percutaneous cholecystostomy versus gallbladder aspiration for acute cholecystitis: A prospective randomized controlled trial. AJR Am J Roentgenol 2004; 183: 193–6. 15208137 10.2214/ajr.183.1.1830193

[4] Mori Y , Itoi T , Baron TH *et al*. Tokyo Guidelines 2018: Management strategies for gallbladder drainage in patients with acute cholecystitis (with videos). J Hepatobiliary Pancreat Sci 2018; 25: 87–95. 28888080 10.1002/jhbp.504

[5] Siddiqui A , Kunda R , Tyberg A *et al*. Three‐way comparative study of endoscopic ultrasound‐guided transmural gallbladder drainage using lumen‐apposing metal stents versus endoscopic transpapillary drainage versus percutaneous cholecystostomy for gallbladder drainage in high‐risk surgical patients with acute cholecystitis: Clinical outcomes and success in an International. Multicenter Study Surg Endosc 2019; 33: 1260–70. 30209610 10.1007/s00464-018-6406-7

[6] Tyberg A , Saumoy M , Sequeiros EV *et al*. EUS‐guided versus percutaneous gallbladder drainage: Isn't it time to convert? J Clin Gastroenterol 2018; 52: 79–84. 28009687 10.1097/MCG.0000000000000786

[7] Itoi T , Sofuni A , Itokawa F *et al*. Endoscopic transpapillary gallbladder drainage in patients with acute cholecystitis in whom percutaneous transhepatic approach is contraindicated or anatomically impossible (with video). Gastrointest Endosc 2008; 68: 455–60. 18561927 10.1016/j.gie.2008.02.052

[8] McLoughlin RF , Patterson EJ , Mathieson JR , Cooperberg PL , MacFarlane JK . Radiologically guided percutaneous cholecystostomy for acute cholecystitis: Long‐term outcome in 50 patients. Can Assoc Radiol J 1994; 45: 455–9. 7982107

[9] Andren‐Sandberg A , Haugsvedt T , Larssen TB , Sondenaa K . Complications and late outcome following percutaneous drainage of the gallbladder in acute calculous cholecystitis. Dig Surg 2001; 18: 393–8. 11721115 10.1159/000050180

[10] Granlund A , Karlson BM , Elvin A , Rasmussen I . Ultrasound‐guided percutaneous cholecystostomy in high‐risk surgical patients. Langenbecks Arch Surg 2001; 386: 212–7. 11382324 10.1007/s004230100211

[11] Sagami R , Hayasaka K , Ujihara T *et al*. Endoscopic transpapillary gallbladder drainage for acute cholecystitis is feasible for patients receiving antithrombotic therapy. Dig Endosc 2020; 32: 1092–9. 32052507 10.1111/den.13650

[12] Itoi T , Kawakami H , Katanuma A *et al*. Endoscopic nasogallbladder tube or stent placement in acute cholecystitis: A preliminary prospective randomized trial in Japan (with videos). Gastrointest Endosc 2015; 81: 111–8. 25527052 10.1016/j.gie.2014.09.046

[13] Itoi T , Coelho‐Prabhu N , Baron TH . Endoscopic gallbladder drainage for management of acute cholecystitis. Gastrointest Endosc 2010; 71: 1038–45. 20438890 10.1016/j.gie.2010.01.026

[14] Ogawa O , Yoshikumi H , Maruoka N *et al*. Predicting the success of endoscopic transpapillary gallbladder drainage for patients with acute cholecystitis during pretreatment evaluation. Can J Gastroenterol 2008; 22: 681–5. 18701945 10.1155/2008/702516 PMC2661289

[15] Yane K , Maguchi H , Katanuma A *et al*. Feasibility, efficacy, and predictive factors for the technical success of endoscopic nasogallbladder drainage: A prospective study. Gut Liver 2015; 9: 239–46. 25287172 10.5009/gnl14070 PMC4351032

[16] Maruta A , Iwata K , Iwashita T *et al*. Factors affecting technical success of endoscopic transpapillary gallbladder drainage for acute cholecystitis. J Hepatobiliary Pancreat Sci 2020; 27: 429–36. 32352636 10.1002/jhbp.744

[17] Sato J , Nakahara K , Michikawa Y *et al*. Clinical outcomes and predictors of technical failure of endoscopic transpapillary gallbladder drainage in acute cholecystitis. Scand J Gastroenterol 2023; 58: 286–90. 36069161 10.1080/00365521.2022.2118554

[18] Hirakawa N , Yamamoto K , Sofuni A *et al*. Factors predicting technical failure of endoscopic transpapillary gallbladder drainage for acute cholecystitis. DEN Open 2024; 4: e308. 37915764 10.1002/deo2.308 PMC10616688

[19] Sagami R , Hayasaka K , Ujihara T *et al*. A new technique of endoscopic transpapillary gallbladder drainage combined with intraductal ultrasonography for the treatment of acute cholecystitis. Clin Endosc 2020; 53: 221–9. 31684701 10.5946/ce.2019.099 PMC7137567

[20] Yoshida M , Naitoh I , Hayashi K *et al*. Four‐Step Classification of Endoscopic Transpapillary Gallbladder Drainage and the Practical Efficacy of Cholangioscopic Assistance. Gut Liver 2021; 15: 476–85. 33402544 10.5009/gnl20238 PMC8129659

[21] Nakahara K , Michikawa Y , Morita R *et al*. Endoscopic transpapillary gallbladder drainage using the balloon occlusion method to advance the guidewire into the cystic duct. Endoscopy 2020; 52: E339–E41. 32187629 10.1055/a-1125-5826

[22] Nakahara K , Michikawa Y , Sato J *et al*. Double‐guidewire technique for endoscopic transpapillary gallbladder stenting. J Hepatobiliary Pancreat Sci 2022; 29: e50–e1. 35037414 10.1002/jhbp.1114

[23] Mandai K , Yoshimoto T . Cystic duct straightening using a fine‐gauge balloon dilator for successful endoscopic transpapillary gallbladder stenting. J Hepatobiliary Pancreat Sci 2023; 30: e62–e3. 36660798 10.1002/jhbp.1305

[24] Bang JY , Arnoletti JP , Wagner A , Varadarajulu S . EUS‐guided gallbladder drainage in acute cholecystitis: Long‐term problems with surgical approach. Gut 2024; 73: 395–7. 38050116 10.1136/gutjnl-2023-331245 PMC10894811

[25] Grover D , Fatima I , Dharan M . Comparison of trans‐gastric versus trans‐enteric (trans‐duodenal or trans‐jejunal) endoscopic ultrasound guided gallbladder drainage using lumen apposing metal stents. World J Gastrointest Endosc 2023; 15: 574–83. 37744320 10.4253/wjge.v15.i9.574 PMC10514705

[26] Perez‐Miranda M . Technical considerations in EUS‐guided gallbladder drainage. Endosc Ultrasound 2018; 7: 79–82. 29667620 10.4103/eus.eus_5_18 PMC5914190

[27] Walter D , Teoh AY , Itoi T *et al* EUS‐guided gall bladder drainage with a lumen‐apposing metal stent: A prospective long‐term evaluation. Gut 2016; 65: 6–8. 26041748 10.1136/gutjnl-2015-309925

[28] Anderloni A , Buda A , Vieceli F , Khashab MA , Hassan C , Repici A . Endoscopic ultrasound‐guided transmural stenting for gallbladder drainage in high‐risk patients with acute cholecystitis: A systematic review and pooled analysis. Surg Endosc 2016; 30: 5200–8. 27059975 10.1007/s00464-016-4894-x

[29] Dollhopf M , Larghi A , Will U *et al*. EUS‐guided gallbladder drainage in patients with acute cholecystitis and high surgical risk using an electrocautery‐enhanced lumen‐apposing metal stent device. Gastrointest Endosc 2017; 86: 636–43. 28259594 10.1016/j.gie.2017.02.027

[30] Martinez‐Moreno B , Lopez‐Roldan G , Martinez‐Sempere J , de‐Madaria E , Jover R , Aparicio JR . Long‐term results after EUS gallbladder drainage in high‐surgical‐risk patients with acute cholecystitis: A 3‐year follow‐up registry. Endosc Int Open 2023; 11: E1063–E8. 37954111 10.1055/a-2180-9817 PMC10637859

[31] Cho SH , Oh D , Song TJ *et al*. Comparison of the effectiveness and safety of lumen‐apposing metal stents and anti‐migrating tubular self‐expandable metal stents for EUS‐guided gallbladder drainage in high surgical risk patients with acute cholecystitis. Gastrointest Endosc 2020; 91: 543–50. 31629721 10.1016/j.gie.2019.09.042

[32] Lisotti A , Linguerri R , Bacchilega I , Cominardi A , Marocchi G , Fusaroli P . EUS‐guided gallbladder drainage in high‐risk surgical patients with acute cholecystitis‐procedure outcomes and evaluation of mortality predictors. Surg Endosc 2022; 36 (1): 569–78. 33507383 10.1007/s00464-021-08318-z PMC7842173

[33] Teoh AYB , Kitano M , Itoi T *et al*. Endosonography‐guided gallbladder drainage versus percutaneous cholecystostomy in very high‐risk surgical patients with acute cholecystitis: An international randomised multicentre controlled superiority trial (DRAC 1). Gut 2020; 69: 1085–91. 32165407 10.1136/gutjnl-2019-319996

[34] Iino C , Shimoyama T , Igarashi T *et al*. Comparable efficacy of endoscopic transpapillary gallbladder drainage and percutaneous transhepatic gallbladder drainage in acute cholecystitis. Endosc Int Open 2018; 6: E594–601. 29744378 10.1055/s-0044-102091 PMC5940465

[35] Mu P , Lin Y , Zhang X *et al*. The evaluation of ENGBD versus PTGBD in high‐risk acute cholecystitis: A single‐center prospective randomized controlled trial. EClinicalMedicine 2021; 31: 100668. 33385126 10.1016/j.eclinm.2020.100668 PMC7772541

[36] Conway JD , Russo MW , Shrestha R . Endoscopic stent insertion into the gallbladder for symptomatic gallbladder disease in patients with end‐stage liver disease. Gastrointest Endosc 2005; 61: 32–6. 15672053 10.1016/s0016-5107(04)02445-9

[37] Hatanaka T , Itoi T , Ijima M *et al*. Efficacy and safety of endoscopic gallbladder stenting for acute cholecystitis in patients with concomitant unresectable cancer. Intern Med 2016; 55: 1411–7. 27250045 10.2169/internalmedicine.55.5820

[38] Kedia P , Sharaiha RZ , Kumta NA *et al*. Endoscopic gallbladder drainage compared with percutaneous drainage. Gastrointest Endosc 2015; 82: 1031–6. 25952093 10.1016/j.gie.2015.03.1912

[39] Inoue T , Okumura F , Kachi K *et al*. Long‐term outcomes of endoscopic gallbladder stenting in high‐risk surgical patients with calculous cholecystitis (with videos). Gastrointest Endosc 2016; 83: 905–13. 26364963 10.1016/j.gie.2015.08.072

[40] Maruta A , Iwashita T , Iwata K *et al*. Permanent endoscopic gallbladder stenting versus removal of gallbladder drainage, long‐term outcomes after management of acute cholecystitis in high‐risk surgical patients for cholecystectomy: Multi‐center retrospective cohort study. J Hepatobiliary Pancreat Sci 2021; 28: 1138–46. 33844472 10.1002/jhbp.967

[41] Sato J , Nakahara K , Michikawa Y *et al*. Long‐term outcomes of endoscopic transpapillary gallbladder drainage using a novel spiral plastic stent in acute calculus cholecystitis. BMC Gastroenterol 2022; 22: 539. 36564715 10.1186/s12876-022-02610-5 PMC9784005

[42] Inoue T , Suzuki Y , Yoshida M *et al*. Long‐term impact of endoscopic gallbladder stenting for calculous cholecystitis in poor surgical candidates: A multi‐center comparative study. Dig Dis Sci 2023; 68: 1529–38. 35989382 10.1007/s10620-022-07651-0

[43] Hayat U , Al Shabeeb R , Perez P *et al*. Safety and adverse events of EUS‐guided gallbladder drainage using lumen‐apposing metal stents and percutaneous cholecystostomy tubes: A systematic review and meta‐analysis. Gastrointest Endosc 2024; 99: 444–8.e1. 37871846 10.1016/j.gie.2023.10.043

[44] Sagami R , Mizukami K , Sato T , Nishikiori H , Murakami K . Strategy comparison of endoscopic ultrasound‐guided gallbladder drainage to percutaneous transhepatic gallbladder drainage, following failed emergent endoscopic transpapillary gallbladder drainage. J Clin Med 2023; 12: 7034. 10.3390/jcm12227034 PMC10671954 38002649

[45] Luk SW , Irani S , Krishnamoorthi R , Wong Lau JY , Wai Ng EK , Teoh AY . Endoscopic ultrasound‐guided gallbladder drainage versus percutaneous cholecystostomy for high risk surgical patients with acute cholecystitis: A systematic review and meta‐analysis. Endoscopy 2019; 51: 722–32. 31238375 10.1055/a-0929-6603

[46] Hemerly MC , de Moura DTH , do M *et al*. Endoscopic ultrasound (EUS)‐guided cholecystostomy versus percutaneous cholecystostomy (PTC) in the management of acute cholecystitis in patients unfit for surgery: A systematic review and meta‐analysis. Surg Endosc 2023; 37: 2421–38. 36289089 10.1007/s00464-022-09712-x

[47] Nishiguchi K , Ogura T , Okuda A *et al*. Endoscopic gallbladder drainage for acute cholecystitis with high‐risk surgical patients between transduodenal and transpapillary stenting. Endosc Ultrasound 2021; 10: 448–54. 34782492 10.4103/EUS-D-20-00130 PMC8785679

[48] Inoue T , Yoshida M , Suzuki Y *et al*. Comparison of the long‐term outcomes of EUS‐guided gallbladder drainage and endoscopic transpapillary gallbladder drainage for calculous cholecystitis in poor surgical candidates: A multicenter propensity score‐matched analysis. Gastrointest Endosc 2023; 98: 362–70. 37059367 10.1016/j.gie.2023.04.002

[49] Krishnamoorthi R , Jayaraj M , Thoguluva Chandrasekar V *et al*. EUS‐guided versus endoscopic transpapillary gallbladder drainage in high‐risk surgical patients with acute cholecystitis: A systematic review and meta‐analysis. Surg Endosc 2020; 34: 1904–13. 32048019 10.1007/s00464-020-07409-7

[50] Rerknimitr R , Pham KC . Practical approaches for high‐risk surgical patients with acute cholecystitis: The percutaneous approach versus endoscopic alternatives. Clin Endosc 2020; 53: 678–85. 31914724 10.5946/ce.2019.186 PMC7719420

[51] Mohan BP , Khan SR , Trakroo S *et al*. Endoscopic ultrasound‐guided gallbladder drainage, transpapillary drainage, or percutaneous drainage in high risk acute cholecystitis patients: A systematic review and comparative meta‐analysis. Endoscopy 2020; 52: 96–106. 31645067 10.1055/a-1020-3932

[52] Saumoy M , Yang J , Bhatt A *et al*. Endoscopic therapies for gallbladder drainage. Gastrointest Endosc 2021; 94: 671–84. 34344541 10.1016/j.gie.2021.05.031

[53] Podboy A , Yuan J , Stave CD , Chan SM , Hwang JH , Teoh AYB . Comparison of EUS‐guided endoscopic transpapillary and percutaneous gallbladder drainage for acute cholecystitis: A systematic review with network meta‐analysis. Gastrointest Endosc 2021; 93: 797–804 e1. 32987004 10.1016/j.gie.2020.09.040

